# Special Issue on Advanced Mechanical Modeling of Nanomaterials and Nanostructures

**DOI:** 10.3390/nano12132291

**Published:** 2022-07-04

**Authors:** Rossana Dimitri, Francesco Tornabene

**Affiliations:** Department of Innovation Engineering, Università del Salento, Via per Monteroni, 73100 Lecce, Italy

The increased requirements in design and manufacturing nanotechnology have favored the development of enhanced composite materials with tailored properties, such as functionally graded (FG) and carbon-based materials, primarily carbon nanotubes (CNTs), and graphene sheets or nanoplatelets, because of their remarkable mechanical properties, electrical conductivity, and high permeability. In such a context, nanoscaled structural elements such as nanobeams and nanoplates are being widely adopted as key components in different modern engineering devices, including sensors, actuators, nanoelectromechanical systems (NEMS), transistors, probes, among others. The complicated nature of similar structural systems requires a proper investigation of their fundamental properties, from an experimental, theoretical, and computational perspective. In line with the experimental findings, classical continuum theories are unable to interpret realistically the physical and mechanical properties of nanomaterials and nanostructures, whereas nonlocal formulations are more prone to explore their possible size-dependence in most static, dynamic, fracture mechanics problems.

This Special Issue has collected 11 papers on the application of high-performing computational strategies and enhanced theoretical formulations to solve a wide variety of linear or nonlinear problems in a multiphysical sense, together with different experimental evidences. Thus, classical and nonclassical theories have been proposed and compared together with multiscale approaches and homogenization techniques for different structural members and practical examples.

More specifically, the first paper authored by M. Soltani et al. [1] leverages a Vlasov thin-walled beam theory and energy method to study the nonlocal flexural-torsional stability of FG tapered thin-walled beam-columns, accounting for the coupled interaction among axial and bending forces. The differential quadrature method (DQM) is selected by the authors as an efficient numerical strategy to solve the coupled governing equations of the problem, checking for the sensitivity of the response for different input parameters, such as the power-law index, nonlocal parameter, axial load eccentricity, mode number and tapering ratio, as useful for design purposes. In the further work by A.H. Sofiyev et al. [2], a Donnel-type shell theory and first order shear deformation assumptions are applied to assess the buckling behavior of FG-CNT reinforced composite conical structures under a combined axial/lateral or axial/hydrostatic loading condition. The governing equations of the problem are transformed into algebraic equations using the Galerkin procedure, while providing an analytical expression for the critical value of the combined loading, as useful for different practical engineering systems and devices.

In the same setting, several works from the literature have recently focused on structures embedding FG materials, even with possible defects and porosities, and have developed innovative analytical and numerical models combined with different higher-order assumptions for many multiphysical problems [3,4,5,6,7] and multiscale electromechanical applications [8,9,10]. With the continuous application of carbon nanomaterials, modified graphene materials doped with epoxy resin have become a key topic for the design and development of novel epoxy composites. In such a context, the molecular dynamics (MD) simulation technology has been successfully applied in the work by Q. Duan et al. [11] for the evaluation of the effect of a fluorinated graphene oxide layer spacing on the thermo-mechanical properties of fluorinated epoxy resins (F-EP). In this paper, it is found that a fluorinated graphene oxide (FGO) with ordered filling can significantly improve the thermo-mechanical properties of F-EP, and the modification effect is better than that of a FGO with a disordered filling. The same computational tool is, moreover, applied by X. Song et al. [12] for the computational study of the surface bonding based on nanocone arrays, accounting for the effects of separation distance, contact length, temperature and size of cones. The main findings from [12] have revealed to be useful in designing advanced metallic bonding processes at low temperatures and pressure with tenable performances. In line with the previous two works, in the work authored by B. Yang et al. [13], the MD is applied to study the thermal stability of the nanotwinned diamond and synthesized nanotwinned cubic boron nitride for nanotwinned structures with enhanced mechanical properties.

Today, graphene nanoribbons (GNR)-based materials represent a valid alternative for the reinforcement of nanoelectronics structures, due to their outstanding properties. Their synthesis process, however, cannot avoid the presence of possible defects such as Stone–Wales (SW), extended line of defects (ELD), and nanopores that can compromise the integrity of structures. This is gradually leading to an increased amount of works focusing on the effect of defects and edges on the mechanical properties of GNR, primarily applying MD simulations [14,15,16]. Among coupled problems, the work by S.K. Jena et al. [17] provides a useful investigation on the buckling behavior of Euler–Bernoulli nanobeams immersed within an electro-magnetic field, based on the Eringen’s nonlocal assumptions and Rayleigh-Ritz method. The authors propose shifted Chebyshev polynomials to avoid any ill-conditioning of the system for a higher number of terms in the approximation due to the orthogonality of functions.

Among the large number of materials suitable for practical applications in tissue engineering, silk fibroin (SF) obtained from Bombyx mori silkworms is largely applied as a scaffold material due to its excellent biocompatibility and low immune reaction [18,19]. As the main factors affecting the mechanical properties of fibrous membranes are the micro-structure and single fiber properties of fibrous membranes, in the work [20], the authors establish the uniaxial tensile force relationship between single fibers and fibrous membranes by means of a micro-mechanical approach. The applicability of the analytical model proposed in [20] is evaluated comparatively against the experimental predictions from the literature. A different multi-scale mechanics concept is applied in [21], combined to a classical finite element approach, to assess the tensile behavior in polymers with randomly oriented and agglomerated cellulose nanofibers, while exploring the property interactions across different length scales.

At the same time, the extensive use of piezoelectric-based pressure sensors for tactile sensing applications has increased the sensibility of the scientific community versus polystyrene in lieu of polymer/composite or crystal materials due to its low-cost fabrication method and large area fabrication. In this context, the work authored by T.S. Ramadoss et al. [22] proposes an inexpensive atactic polystyrene as base polymer and fabricate functional fibers, and an electrospinning method is used. The authors study experimentally the fiber morphologies by using a field-emission scanning electron microscope, while proposing a unique pressure sensor fabrication method. In the last Ref. [23], some possible industrial applications of nanomaterials obtained from the waste ashes are suggested, including, for example, inks for Aerosol Jet^®^ Printing, with useful insights from a sustainable perspective.

Although this Special Issue has been closed, further developments on the theoretical and computational modeling of enhanced nanocomposite materials and structures are expected, including their static, dynamic, and buckling responses, as well as their fracture mechanics, and these will be useful for many industrial applications.
